# Supplementary material for: Gender perspectives on zoonotic disease epidemiology; A strength weakness opportunities threats analysis in Bundibugyo district, Uganda
Source: PLoS One. 2025 May 29;20(5):e0324442. doi: 10.1371/journal.pone.0324442 (PMC12122033; doi:10.1371/journal.pone.0324442)
Supplement: S1 Data — (PDF) [file pone.0324442.s001.pdf]

Consent forms for data collection

Title: Bat ecology and social epidemiology of filoviridae haemorrhagic fevers in Uganda.

Introduction

Hello Sir/Madam, my name is……………………………………………., and I am part of a PhD student from Makerere University College of veterinary medicine, Biosecurity and Animal resources conducting a study to assess the bat ecology and social epidemiology of filoviridae haemorrhagic fevers in Uganda.

Purpose of the study

Pathogen circulation in reservoir hosts is an essential precursor to spillover, but its ecological aspects are often poorly understood relative to post-spillover processes. Bats are an important clade to study, as they host a uniquely rich set of viruses, including many important emerging zoonotic filoviruses. This study is aimed at elucidating the ecological determinants that favour maintenance/presence of filoviruses at the human-bat interface, and the subsequent processes leading to infection and virus shedding.

Procedure

The study is to be will aim at accelerating the level of introduction of filovirus from feacal matter, bat behaviour and bat distribution, anthropogenic factors and modelling filoviruses distribution to determine the frequency of their introduction into the human population. Cross sectional study design will be used for this research. Questionnaires, Key informant guides, field sample collection and in-depth interview guides will be used to collect the data. The interview will take approximately 20 to 30 minutes.

Potential benefits

Results from this study will inform about previous spillover events of filoviruses in the Rwenzori areas including Bundibugyo, Mubende and Kassanda districts to raise awareness on environmental factors affecting bats ecology, their environmental reservoirs and social epidemiological dynamics by providing scientific evidence for policy and public health strategy development against bat-borne zoonotic illness outbreaks in Uganda.

Potential risks and discomfort: There will be minimal risks or discomfort from participating in this study.

Voluntary participation: Participation in this study is voluntary and will not affect you. If you don't wish to participate in this discussion, you can ask to be excused. Even as you answer questions asked to you, you can decide to refuse to answer any question if you do not feel comfortable to do so.

Confidentiality: All collected information from this interview will be kept confidential. During the discussions, we shall not use your names but rather assign you with numbers to further protect

your privacy. The collected data will also only be accessible to the study investigators and will be kept in a secure place.

Compensation: For the time of participation in this study we will provide you with ten thousand Ugandan shillings (10,000UGX).

Question: Do you have any questions about the study that you would like me to attend to?

Contact information.

In case you have any other questions or concerns about this study later, you may contact Ms. Ninsiima Lesley Rose the Principal Investigator of the survey at telephone numbers: 0785254323 or 0703186003 and Email: lninsiima04@gmail.com.

If you have any questions about your rights as a participant or you feel that you have been harmed by taking part in the study, please contact Mr. Paul Kutyabami, the Chairperson of the School of Makerere University College of Health Sciences Research and Ethics Committee Email: paulkutyabami@yahoo.com.

Thank you.

Statement of informed consent

I would now like to get your consent to participate before I proceed. Do you consent to participate in the study

Respondent response: Yes or No

I understand the procedures described above. My questions have been answered to my satisfaction and I agree to participate in the study.

Signature /thumb print of participant……………………. Date…………………………

Signature/thumbprint of witness………………………….. Date…………………………

Signature of the interviewer………………………………… Date…………………………
