# Supplementary material for: Gender perspectives on zoonotic disease epidemiology; A strength weakness opportunities threats analysis in Bundibugyo district, Uganda
Source: PLoS One. 2025 May 29;20(5):e0324442. doi: 10.1371/journal.pone.0324442 (PMC12122033; doi:10.1371/journal.pone.0324442)
Supplement: S1 File — (DOCX) [file pone.0324442.s002.docx]

**Supplementary 1: Qualitative tool for Key Informants, In-depth interviews and Focus Group Discussions**

**Key Informant Interviews (KIIs) Questionnaire**

1. Can you tell me a bit about your role in the community and how long you have been working in Bundibugyo? (*Probe how you engage with communities regarding health issues?*)
2. What zoonotic diseases are most commonly found in Bundibugyo district? (*Probe: Are there any specific zoonotic diseases that you believe are more prevalent in certain areas or among certain groups?*)
3. How does your organization or role help in the identification and prevention of zoonotic diseases? (*Probe*: *What strategies do you think are most effective in educating the community?*)
4. What do you think are the primary threats increasing the risk of zoonotic disease spillover in Bundibugyo? (*Probe: How does land encroachment, hunting, or animal movement contribute to this risk?*)
5. How do cultural practices, such as consuming wild animals, affect the transmission of zoonotic diseases in the district? (*Probe*: *Have you seen an increase in certain diseases linked to these practices?*)
6. In your opinion, what weaknesses exist in the community that make it vulnerable to zoonotic diseases? (*Probe*: *Are there specific knowledge gaps or cultural practices that hinder disease prevention?*)
7. How would you assess the community's preparedness for zoonotic disease outbreaks? (*Probe*: *Are there any existing systems for early detection that need strengthening?)*
8. What strengths does the community possess in managing zoonotic diseases? (*Probe*: *How do local health workers, village leaders, or cultural leaders play a role in disease prevention?)*
9. Can you describe any community-driven initiatives or health strategies that have been successful in reducing zoonotic disease risk? (*Probe*: *What role do women and men play in these initiatives?)*
10. What opportunities exist for improving zoonotic disease prevention and control in Bundibugyo?
    *(Probe*: *Are there gaps in health services, and how can they be addressed?)*
11. How can local government, NGOs, or community organizations contribute to strengthening disease prevention? (*Probe*: *What kind of training or resources are needed by health workers?*)
12. What urgent steps do you recommend to prevent zoonotic disease outbreaks in the district? (*Probe*: *Are there specific policy changes or strategies that need to be implemented?)*
13. Is there anything else you'd like to add regarding zoonotic disease management in Bundibugyo?

**In-Depth Interviews (IDIs) Questionnaire**

1. How long have you lived in Bundibugyo district? (*Probe*: *What has been your involvement in the community, especially regarding health matters?*)
2. Can you name some zoonotic diseases that you know affect Bundibugyo? (*Probe*: *How did you first hear about these diseases?*)
3. Do you feel that the community is aware of the risks associated with zoonotic diseases? (*Probe*: *What kinds of information do people usually share about these diseases?*)
4. What activities or practices in your community do you think increase the risk of zoonotic diseases? (*Probe*: *How does hunting, animal grazing, or cultural practices impact the spread of diseases?)*
5. How do you think political instability or climate change contributes to the spread of zoonotic diseases in Bundibugyo? (*Probe*: *Have you noticed any patterns of disease outbreaks linked to these factors?*)
6. What challenges do you think the community faces in preventing or controlling zoonotic diseases? (*Probe*: *Is there a lack of resources or health knowledge?)*
7. How does the community respond to outbreaks of zoonotic diseases, and what gaps in response do you observe? (*Probe*: *Is there any confusion about how to prevent diseases?*)
8. Can you share some examples of how the community has managed zoonotic diseases successfully in the past? (*Probe*: *How do women, men, and children participate in these efforts?*)
9. How does the community engage with health services, and what has been the impact of these services in controlling zoonotic diseases? (*Probe*: *Have you seen improvements in local health services over time?*)
10. What do you think can be done to improve the community’s response to zoonotic diseases?
    *(Probe*: *Are there local health workers or organizations that can assist more?*)
11. How can awareness about zoonotic diseases be spread more effectively in Bundibugyo? (*Probe*: *What role do community meetings, radio stations, or other media play in this?*)
12. What do you think is the most important thing that should be done right now to prevent zoonotic diseases? (*Probe*: *What specific actions can be taken at the community level?*)
13. Do you have any recommendations on how to improve education and communication about zoonotic diseases in your community?

**Focus Group Discussions (FGDs) Questionnaire**

1. Can everyone introduce themselves and share how long they've lived in Bundibugyo? (*Probe*: *What is your role within the community?*)
2. What are some of the zoonotic diseases that you know of in this area? (*Probe*: *Which diseases are you most worried about? Why?*)
3. Do you think everyone in the community knows about these diseases? (*Probe*: *Are there certain diseases that people tend to ignore or not discuss?*)
4. What practices in your community do you think increase the risk of zoonotic diseases? (*Probe*: *How does hunting, grazing, or cultural food practices affect the spread of diseases?*)
5. Do you think that climate change or land encroachment is making things worse? (*Probe*: *How does this affect the local wildlife and human-animal interactions?*)
6. What do you think are the weaknesses in your community when it comes to managing zoonotic diseases? (*Probe*: *Do you think people understand how to prevent these diseases?*)
7. How can the community improve hygiene and sanitation to reduce the spread of diseases? (*Probe*: *Who is responsible for ensuring cleanliness at the household level?*)
8. Can you think of any successful efforts the community has taken to manage zoonotic diseases? (*Probe*: *How did these efforts involve both men and women?*)
9. How can the community work together to fight zoonotic diseases? (*Probe*: *How do you think women can help in disease prevention?*)
10. What resources or opportunities would help the community better manage zoonotic diseases? (*Probe*: *How can health workers, leaders, or NGOs support your community?*)
11. What strategies can improve awareness about zoonotic diseases? (*Probe*: How can traditional leaders or community groups help spread information?)
12. If you had to pick one thing to improve in the community to reduce zoonotic diseases, what would it be? (*Probe*: *What practical steps can be taken in the short term?*)
13. Do you have any final thoughts or suggestions on how to handle zoonotic diseases in Bundibugyo?
