# Supplementary material for: Gender perspectives on zoonotic disease epidemiology; A strength weakness opportunities threats analysis in Bundibugyo district, Uganda
Source: PLoS One. 2025 May 29;20(5):e0324442. doi: 10.1371/journal.pone.0324442 (PMC12122033; doi:10.1371/journal.pone.0324442)
Supplement: Attachment — Submitted filename: rebuttal.docx [file pone.0324442.s006.docx]

Makerere University

School of biosecurity, ecosystems and veterinary Public Health

26^th^ July 2024

To The Editor

Plos One

We are grateful to the editor for giving us the opportunity to edit our manuscript titled “Gender perspectives on zoonotic disease epidemiology; A Strength Weakness Opportunities Threats analysis in Bundibugyo district, Uganda.” We also appreciate the peer reviewers’ efforts for their insights, which have significantly improved the quality of our manuscript.

We have addressed the reviewers’ comments to the best of our ability, hence would like to submit the revised manuscript.

| **Comment** | **Response** |
| --- | --- |
| **Editor** |  |
| Modify the figure 1. I advise you to use freely accessible layers and Geographic Information System software to generate your own map showing precise study sites. | This has been edited to highlight the district of study |
| Line 202-203: Where is the final list of prioritized zoonoses | All zoonoses highlighted were mentioned by participants as diseases that are of priority to them. |
| 375 – 387: What is the relevance of this paragraph to your study findings? | This shows the gender roles men and women play in prevention of zoonotic diseases from both animals and humans and how exposure to this knowledge has helped them. |
| Check for the manuscript for the many typos. Examples are: o Line 125: Missing word in the sentence ending with “Lake Albert to the…” | Thank you for this comment. This has been edited as per the comment given. |
| Line 126: The sentence is incomplete | The sentence has been completed. |
| 196-197: Themes c and d are the same. | This has been edited to include community strength for zoonotic disease management as theme c |
| Line 202: Is Swine fever a zoonoses? Clarify why it was included | This has been removed as per the comment since it’s not a zoonotic disease. |
| 306-307: “Importance of” is repeated twice | Thank you for this comment. This has been edited. |
| **Reviewer 1** |  |
| Authors addressed clearly aspects related on the Gender perspectives on zoonotic disease epidemiology using a SWOT analysis in Bundibugyo a district of Uganda. The methodology is fitting the results presented in the manuscript and the topic is actually relevant for the control of zoonotic diseases in the country an the sub-region.  Therefore some comments are proposed to improve this manuscript:  >Line 73: Better to put a citation (s) for this sentence | The citation has been included. Thank you for the comment. |
| >Line 76: remove the coma at the beginning of the sentence | This has been removed as per the comment. Thank you for the comment. |
| >Line 87: In my opinion, not all men are playing such role except where this gender role can be played, so reformulate the sentence. | Thank you for the comment. This statement has been reformulated. |
| >Line 137: Is it possible to make clear the district of study by extracting it from the map of Uganda. There the borders could be well visible. | Thank you for the comment. The map has been deleted form this article. |
| >147: make a correction on this sentence: All participants mostly understood English. | This has been edited as per the comment given. |
| How about to include also participants who didn't get a formal education, because zoonotic diseases mitigation cannot be limited to knowledge. Illiteracy can maybe play a role of their spread and therefore constitute a weakness for their control. What do you think on this? | Thank you for this comment. Most participants understand selected for this study were found to understand English as well however originally during the selection knowing English was not a selection criterion. |
| >Line 288: diseases | Thank you for the comment. This has been edited to include zoonotic diseases. |
| >Line 310: In this paragraph, the capture on how diseases manifest and traverse along lines of gender is not clearly stated. | Thanks for the comment. This quote was aimed to show how the women prevent the diseases by having the prevention strategies in place to prevent the spread of the zoonotic/infectious diseases. I see gender inclusiveness through prevention from homes. |
| Also, Check if some of the opportunities although mentioned by participants are not likely to be part of the Strength for zoonotic diseases management, prevention and control | The strength for zoonotic disease management, prevention and control is looking at what are the advantages and what is working well within the community. Opportunities for communities in management leaveraged on what prizes are within reach for the community to dwell on. |
| >Line 435: remove the citation in the conclusion | Thank you for the comment. This has been removed. |
| **Reviewer 2** |  |
| There were some unclear sentences with some run-ons, that i've noted in the attachment. Suggestions on improving sentence construction were also made to clarify some statements - examples of questions from the IDI, KII, and FGD would be helpful, if authors will oblige | Thank you for the comment. The tool has been uploaded. |
| - suggest for the authors to proofread the entire report again, as the abstract has an obvious mistake (opportunities mentioned twice, no threats) | Thanks for the comment. The repetition has been edited and all included. |
| **Reviewer 3** |  |
| The study explores gender perspectives on zoonotic disease epidemiology using a (strength, weakness, opportunities and threats) SWOT analysis in Bundibugyo district in Uganda. The study adopts the SWOT analysis. While the authors have made a good effort to do so across the key elements, there is need for further clarity within each SWOT element in the discussion session. This should be done by highlighting the gendered aspects in each element e.g. in discussing a strength, discuss how it is a strength for men, for women and why. The authors should also look within the uploaded document and address specific comments mentioned. | Thank you for the comment. This has been highlighted in the whole discussion how it is gender related within each SWOT. All comments have been addressed and worked on. |

Yours Sincerely

Clovice Kankya
