# Supplementary material for: Gender perspectives on zoonotic disease epidemiology; A strength weakness opportunities threats analysis in Bundibugyo district, Uganda
Source: PLoS One. 2025 May 29;20(5):e0324442. doi: 10.1371/journal.pone.0324442 (PMC12122033; doi:10.1371/journal.pone.0324442)
Supplement: Attachment — Submitted filename: rebuttal_auresp_2.docx [file pone.0324442.s007.docx]

Makerere University

School of biosecurity, ecosystems and veterinary Public Health

10^th^ April 2024

To The Editor

Plos One

We are grateful to the editor for giving us the opportunity to edit our manuscript titled “Gender perspectives on zoonotic disease epidemiology; A Strength Weakness Opportunities Threats analysis in Bundibugyo district, Uganda.” We also appreciate the peer reviewers’ efforts for their insights, which have significantly improved the quality of our manuscript.

We have addressed the reviewers’ comments to the best of our ability, hence would like to submit the revised manuscript.

| **Comment** | **Response** |
| --- | --- |
| **Reviewer #1** |  |
| Authors touched a topic addressing the Gender perspectives on zoonotic disease epidemiology; A Strength Weakness  Opportunities Threats analysis in Bundibugyo district, Uganda. They responded correctly all the comments from the reviewers. | Thank you for the feedback, we are really grateful. |
| **Reviewer #4** |  |
| The authors of the manuscript entitled: Gender perspectives on zoonotic disease epidemiology; A Strength Weakness Opportunities Threats analysis in Bundibugyo district, Uganda, have improved the presentation of the work by addressing all comments from reviewers.  This paper would have been beneficial if a list of zoonotic diseases mentioned by the respondents are provided before the ranking? | Thank you for the comment, these have been included in the manuscript. |
| Whereas SWOT approach was used, it not very clear how it was deployed across KIIs, In-depth interviews and FGDs (This could be provided as supplementary files including checklists that were used). It was not clear on who conducted different interviews, and which language was used. | Thank you for the comment. The tool has been included a supplementary material for each of the categories of KIIs, IDIs and FGDs. |
| In the methods sections, 7 sub-counties were considered in the study area, it is not clear on how the respondents for different categories of interviews were selected in 7- sub-counties (A supplementary table) may be need to clarify the approaches used in the selection of respondents. | This is a great comment. The participants recruitment criteria were already explained in details in the manuscript. |
| Some sections in the discussion seem to divert from the subject matter and introduce most the previously reported assertions | Thanks for the comment. These have been further read and edited |

Yours Sincerely

Clovice Kankya
